# Unveiling the Quest: Crafting an Enzyme-Linked Immunosorbent Assay (ELISA) Technique to Uncover COVID-19 Antibodies

**DOI:** 10.7759/cureus.66659

**Published:** 2024-08-11

**Authors:** Nitin Deshpande, Poonam V Suryawanshi, Srikanth Tripathy

**Affiliations:** 1 Department of Health Sciences, Dr. D. Y. Patil Medical College, Hospital and Research Centre, Dr. D. Y. Patil Vidyapeeth (Deemed to be University), Pune, IND; 2 Department of Health Sciences, Premium Chick Feeds Pvt Ltd, Pune, IND; 3 Department of Immunology, Dr. D. Y. Patil Medical College, Hospital and Research Centre, Dr. D. Y. Patil Vidyapeeth (Deemed to be University), Pune, IND; 4 Department of Infectious Diseases, Dr. D. Y. Patil Medical College, Hospital and Research Centre, Dr. D. Y. Patil Vidyapeeth (Deemed to be University), Pune, IND

**Keywords:** nucleocapsid, spike, sars-cov-2 antibodies, elisa, covid-19

## Abstract

The COVID-19 pandemic, caused by the novel coronavirus SARS-CoV-2, has had a profound impact on global health. Rapid and accurate diagnostic tools are crucial for effective disease control and management. The enzyme-linked immunosorbent assay (ELISA) has emerged as a reliable and widely used method for detecting COVID-19 antibodies in patients, which develop in response to SARS-CoV-2 infection. While the ELISA technique is effective in identifying the presence of antibodies and thus confirming exposure to the virus, its role in predicting the clinical course and severity of the disease is limited. ELISA primarily confirms prior exposure to the virus or vaccination status, but it does not directly correlate antibody levels with the severity or progression of the disease. The variability in clinical outcomes is influenced by factors such as viral load, patient co-morbidities, genetic predispositions, and the timing of the immune response.

ELISA has diverse applications in epidemiology, vaccination assessment, and therapeutic development. It determines antibody prevalence, aids in surveillance, and evaluates vaccine effectiveness and antibody protection duration. ELISA quantitatively measures antibody levels, providing insights into the immune response and treatment efficacy. Challenges include specialized facilities and personnel, cross-reactivity, and false results. Multiplex assays and integration with other diagnostics are future directions. In summary, ELISA is an essential tool in COVID-19 diagnostics, enabling precise assessment of the immune response and contributing to effective strategies.

The development of point-of-care devices that integrate ELISA technology could enable rapid and accessible testing in various settings. Additionally, integrating ELISA with other diagnostic platforms could enhance the overall diagnostic capabilities for COVID-19. Despite challenges, ongoing advancements in ELISA technology, and its integration with other diagnostic approaches, hold promise for further improving COVID-19 diagnostics and management strategies.

## Introduction and background

In the aftermath of the COVID-19 pandemic, there is an urgent need for reliable diagnostic tools to identify antibodies against the SARS-CoV-2 virus [1,2]. The enzyme-linked immunosorbent assay (ELISA) has emerged as a crucial technique in serological testing due to its sensitivity, specificity, and efficiency in detecting antibodies in patient samples [3]. This immune response can be due to either natural infection or vaccination. ELISA is helpful for diagnosis, as it can confirm prior exposure to the virus by detecting specific antibodies, regardless of whether they were generated through natural infection or vaccination, thus providing valuable information for assessing immune status and guiding public health decisions.

This paper explores the development and refinement of an ELISA-based method specifically tailored for detecting COVID-19 antibodies. By delving into the intricacies of ELISA design, from selecting antigens to optimizing the assay, this study aims to clarify the fundamental principles and practical considerations essential for accurate and reproducible antibody detection.

ELISA is a laboratory technique that uses specific antibodies to detect and quantify substances such as proteins, peptides, hormones, and antibodies in biological samples [4]. It operates on the principle of antigen-antibody interaction, where a specific antigen is fixed to a solid surface and detected by antibodies that bind to it. This binding interaction can be visualized by adding an enzyme-linked secondary antibody, which generates a measurable signal [5].

In COVID-19, ELISA has been adapted and fine-tuned for detecting antibodies that develop in response to SARS-CoV-2 infection [6]. During infection, the immune system produces antibodies in response to the virus, which can be detected in patient samples like blood or serum using ELISA [7].

Developing an ELISA for COVID-19 antibody detection involves identifying and selecting viral antigens capable of triggering an immune response in infected individuals [8]. These antigens are applied to the surface of a microplate or similar support. Patient samples containing antibodies against SARS-CoV-2 are then added to the wells of the microplate, allowing the antibodies to bind to the immobilized viral antigens [9]. After removing unbound materials through washing, a secondary antibody linked to an enzyme is introduced. This secondary antibody binds to the patient’s antibodies, forming a complex. The enzyme-linked secondary antibody catalyses a reaction that produces a detectable signal, often a colour change, indicating the presence of SARS-CoV-2 antibodies in the patient sample [10].

ELISA serves various purposes in clinical research and public health. It can determine the prevalence of COVID-19 antibodies in a population, aiding in epidemiological studies and surveillance efforts [11]. ELISA is also useful for evaluating the immune response to vaccination, monitoring the effectiveness of vaccination campaigns, and assessing the duration of antibody protection. Additionally, ELISA contributes to the development and evaluation of therapeutic interventions for COVID-19 [12]. Researchers used ELISA to evaluate how individuals responded immunologically to SARS-CoV-2 infection or vaccination, which was critical in assessing the effectiveness of potential therapies, such as vaccines or monoclonal antibodies [13]. ELISA allowed researchers to quantify antibody levels, providing valuable insights into immune responses and treatment efficacy [14]. As the SARS-CoV-2 pandemic took its toll, research on viral immunopathogenesis accelerated to find appropriate diagnostic methods and effective preventative and therapeutic interventions [15].

Moreover, amid the evolving landscape of viral mutations and immune responses, the reliability of ELISA as a diagnostic tool underscores its importance in public health strategies. This research aims to contribute to the broader discussion on serological testing methods, offering insights into how ELISA can be used to understand the dynamics of immune responses, crucial for comprehending COVID-19 infection and immunity.

## Review

ELISA principles

ELISA is a widely used immunological test for detecting and measuring specific analytes, such as antibodies, in biological samples. It operates on the principle of specific binding between antibodies and antigens [16].

In the context of COVID-19, ELISA is employed to detect and measure antibodies against the SARS-CoV-2 virus [17]. The assay begins by coating a microplate, typically made of plastic or glass, with specific antigens related to SARS-CoV-2. These antigens can include viral proteins, peptides, or other components that can trigger an immune response in infected individuals [18].

Once the microplate is coated, patient serum or plasma samples are added to the plate's wells. If the patient has been infected with SARS-CoV-2 and has developed antibodies against the virus, these antibodies will bind specifically to the antigens fixed on the plate [19].

To remove any unbound materials, the microplate is thoroughly washed. This step ensures that only the specific antibodies bound to the antigens remain on the plate, while non-specific substances are removed [20].

Next, enzyme-conjugated secondary antibodies are added to the microplate. These secondary antibodies are designed to bind specifically to the patient's antibodies that have already been attached to the viral antigens on the plate. The secondary antibodies are labelled with enzymes like horseradish peroxidase (HRP) or alkaline phosphatase [21].

Once the secondary antibodies bind to the patient's antibodies on the plate, an enzymatic reaction is triggered by adding a substrate that the enzyme acts upon. This reaction typically results in a colour change or the generation of a fluorescent or luminescent signal. The intensity of this signal correlates with the quantity of antibodies present in the patient sample [22].

Specialized instruments are used to measure the signal, enabling the determination of the presence and quantity of antibodies against SARS-CoV-2 in the patient sample. This information is crucial for diagnosing past or current infections, assessing the immune response to vaccination, and monitoring antibody levels over time [23].

**Figure 1:**
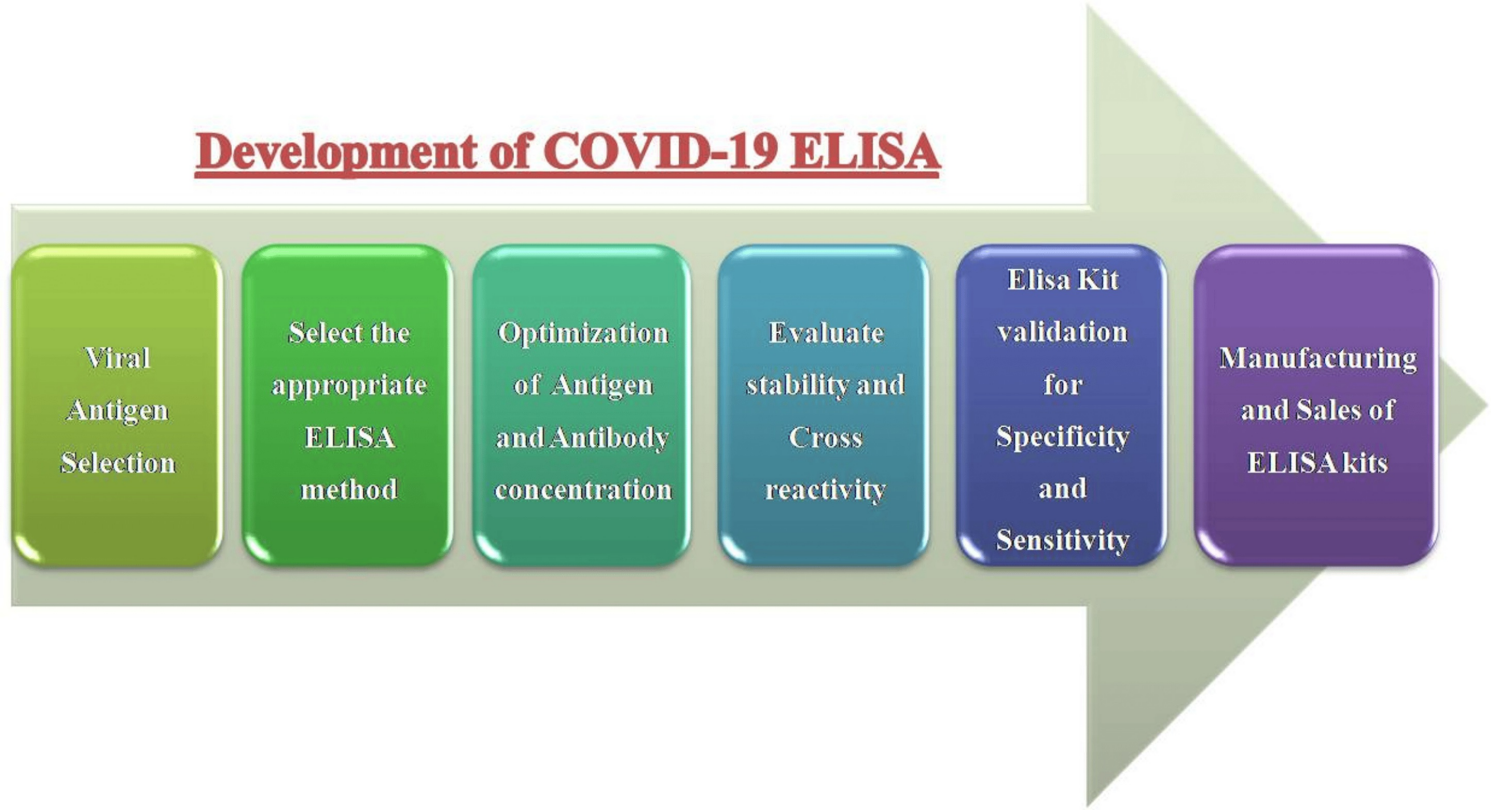
Development of COVID-19 ELISA The illustration depicts the step-by-step procedure to develop a COVID-19 ELISA test. The development process is depicted by a sequence of coloured boxes organized in a linear pattern, with each box indicating a stage in the process. ELISA: Enzyme-linked immunosorbent assay This figure was created in PowerPoint specifically for this manuscript and does not include any reproduced images or content requiring permission. Image credit: Dr. Poonam Suryawanshi

In summary, ELISA is an immunological assay that relies on the specific binding of antibodies to antigens. In the context of COVID-19, ELISA detects and quantifies antibodies against SARS-CoV-2 by coating a microplate with viral antigens, adding patient samples, using enzyme-conjugated secondary antibodies, and measuring the resulting signal [24]. Figure 1 illustrates the process of developing ELISA for COVID-19 antibody detection. This technique has proven to be reliable and widely used in COVID-19 diagnostics and research.

Stages involved in developing a COVID-19 ELISA

In response to the COVID-19 pandemic, developing an effective ELISA for detecting antibodies against SARS-CoV-2 has become crucial. The development process includes several key stages, each essential for ensuring the accuracy, sensitivity, and reliability of antibody detection. Figure 1 outlines the step-by-step process for developing a COVID-19 ELISA test. The development process is represented by a series of coloured boxes arranged in a linear fashion, each box representing a different stage in the process.

Selection of Viral Antigens

The selection of viral antigens is a critical step in developing ELISA assays for COVID-19 detection. Researchers primarily target two key proteins of the SARS-CoV-2 virus: the spike protein (S) and the nucleocapsid protein (N). These proteins play essential roles in the viral life cycle and are immunogenic, triggering an immune response in infected individuals [25].

To coat the microplate in ELISA assays, researchers use recombinant proteins or peptide fragments derived from the spike protein or nucleocapsid protein. Recombinant proteins are produced in the laboratory using genetic engineering techniques, ensuring a consistent and reliable source of antigens. Peptide fragments are short sequences of amino acids that represent specific regions of the viral proteins [26].

The selection of viral antigens is guided by several factors. Researchers aim to identify regions of the spike protein or nucleocapsid protein that are highly immunogenic and capable of eliciting a strong antibody response [27]. By studying the antigenic domains and immunodominant epitopes of SARS-CoV-2, researchers can pinpoint specific regions recognized by antibodies produced during infection [28].

Numerous studies have optimized antigen selection for COVID-19 ELISA assays. These studies involve mapping antigenic domains, identifying immunodominant epitopes targeted by antibodies, and evaluating the binding affinity and specificity of different antigens [29]. Techniques such as peptide scanning, epitope prediction algorithms, and protein-protein interaction studies are used to identify the most suitable antigens for coating the microplate [30].

The goal of antigen selection is to ensure that the ELISA assay can accurately detect and quantify COVID-19 antibodies in patient samples. By using antigens derived from the spike protein or nucleocapsid protein, ELISA assays can specifically target antibodies generated against SARS-CoV-2 [31]. The selected antigens should exhibit high sensitivity and specificity, meaning they can detect true positive cases while minimizing false positive or false-negative results [32].

Assay Formats for COVID-19 ELISA

Assay formats play a crucial role in COVID-19 ELISA for detecting antibodies. Several formats, including indirect, capture, and sandwich ELISA, have been employed to enable the detection of different antibody isotypes and provide flexibility in assay design.

Indirect ELISA: In the indirect ELISA format for COVID-19 antibody detection, the viral antigen is coated onto the microplate. Patient serum or plasma samples are then added to the plate, allowing specific antibodies present in the sample to bind to the viral antigen. After a washing step to remove unbound materials, enzyme-conjugated secondary antibodies, specific to the antibody isotype of interest (e.g., IgM, IgG), are added. These secondary antibodies bind to the patient’s antibodies already bound to the viral antigen [33]. After another washing step to remove unbound secondary antibodies, a substrate specific to the conjugated enzyme is added. The enzyme catalyses a reaction with the substrate, resulting in a measurable signal (e.g., colour change or fluorescence). The intensity of this signal correlates with the presence and quantity of antibodies in the patient sample [34].

Sandwich ELISA: Sandwich ELISA is a format commonly used for the quantitative detection of COVID-19 antibodies. In this format, two different antibodies are utilized: a capture antibody and a detection antibody. The capture antibody is immobilized on the microplate, specifically targeting a different epitope on the viral antigen than the detection antibody. The patient sample, potentially containing viral antigens and antibodies, is then added to the plate [35]. The capture antibody binds the viral antigen present in the sample, while the detection antibody, conjugated to an enzyme, binds to a different epitope on the viral antigen. After washing to remove unbound materials, the addition of a substrate specific to the enzyme generates a measurable signal. The signal intensity reflects the concentration of the viral antigen present in the patient sample [36].

The choice of assay format depends on specific study requirements, including the desired antibody isotype for detection, assay sensitivity and specificity, and availability of reagents. Each format offers advantages and considerations, allowing researchers to tailor the assay design for COVID-19 antibody detection [37].

Optimization of COVID-19 ELISA

Optimization of COVID-19 ELISA assays are crucial steps in ensuring the accuracy and reliability of diagnostic tests. These steps involve fine-tuning various parameters and validating the assay’s specificity and sensitivity against well-characterized reference samples and alternative diagnostic methods [38].

Optimizing assay parameters is essential to maximize COVID-19 ELISA performance. One key factor is determining the optimal antigen concentration for coating the microplate. Different concentrations of viral antigen are tested to identify the concentration that provides the highest signal-to-noise ratio and robust assay performance [39].

Incubation time is another critical parameter requiring optimization. The duration of antigen and patient sample interaction during incubation can influence assay sensitivity. Longer incubation times may enhance antibody binding to the viral antigen, but excessively long incubation times can lead to non-specific binding and increased background noise [30].

Blocking agents are used to prevent non-specific binding of proteins to the microplate surface. The choice of blocking agent, such as bovine serum albumin (BSA) or casein, and the optimal concentration are determined through optimization experiments. This ensures minimal non-specific interactions, improving assay specificity.

Washing conditions are optimized to effectively remove unbound materials while retaining bound antibodies. Factors such as the number of washes, the volume and composition of the washing buffer, and the duration of each wash step are optimized to minimize background noise and maximize signal detection.

Validation of Assay Specificity and Sensitivity

Validation is a critical step in confirming the accuracy and reliability of COVID-19 ELISA assays. The specificity of the assay is assessed by testing well-characterized reference samples, including samples from individuals with known COVID-19 infection and those without the infection, serving as positive and negative controls, respectively. The assay should demonstrate high specificity by accurately identifying positive samples and minimizing false-positive results.

The sensitivity of the assay is determined by assessing its ability to detect low concentrations of antibodies in patient samples. Serial dilutions of reference samples with known antibody concentrations are tested to establish the lower limit of detection. The assay should demonstrate high sensitivity by reliably detecting low antibody concentrations and minimizing false-negative results.

Furthermore, the performance of the COVID-19 ELISA assay is compared with alternative diagnostic methods, such as polymerase chain reaction (PCR)-based assays. This comparative analysis helps validate the assay’s performance against established diagnostic techniques and ensures its reliability in detecting COVID-19 antibodies. Validation studies also involve analysing a statistically significant number of patient samples to evaluate the overall accuracy and performance of the assay. This includes calculating sensitivity, specificity, positive predictive value, and negative predictive value. Commercially available COVID-19 ELISA kits are listed in Table 1. These parameters provide essential information about the assay’s performance in real-world scenarios.

**Table 1:** List of commercially available ELISA kit

Sr. No	Name of company	Name of ELISA kit	Specificity	Sensitivity
1	Euroimmun US Inc., Mountain Lakes, USA	Euroimmun Anti-SARS-COV-2 IgG ELISA	100%	90%
2	Lab-Care Diagnostics (India) Pvt. Ltd., Valsad (Gujarat), India	Novel Corona virus COVID-19 IgG ELISA	98.67% (>97%)	100% (>98%)
3	Transasia Bio-Medicals Ltd., Mumbai (Maharashtra), India	ErbaLisa COVID-19 IgG ELISA	98.1%	98.3%
4	Meril Diagnostics Pvt. Ltd., Vapi (Gujarat), India	Anti-SARS CoV-2 Human IgG ELISA	93%	100%
5	J. Mitra & Co. Pvt. Ltd., Delhi, India	COVID Kawach IgG MICROLISA	100%	96.33%
6	Trivitron Healthcare Pvt. Ltd., Mumbai (Maharashtra), India	COVID KAVACH Anti-SARS CoV-2 Human IgG ELISA kit	100%	98%

Challenges in ELISA implementation

While ELISA has proven to be a valuable tool in COVID-19 diagnostics, it is important to recognize the challenges associated with its implementation and consider future directions for improvement. ELISA helps in treatment strategy by providing crucial information about a patient's immune response. By detecting specific antibodies, ELISA can indicate whether a patient has been previously exposed to the virus or vaccinated, which can influence decisions regarding the necessity and timing of booster vaccinations, the need for continued protective measures, and eligibility for certain treatments, like monoclonal antibodies. Understanding a patient's antibody levels can also aid in monitoring the durability of immunity and tailoring personalized treatment plans to enhance patient outcomes.

These challenges associated with ELISA implementation include the need for specialized laboratory facilities, trained personnel, and the potential for cross-reactivity and false results. To address these limitations, researchers are exploring innovative approaches and technologies to enhance the capabilities of ELISA in COVID-19 diagnostics.

Specialized Laboratory Facilities and Trained Personnel

One challenge with ELISA is the requirement for specialized laboratory facilities equipped with the necessary infrastructure and instruments. ELISA assays often involve multiple steps, including antigen coating, sample preparation, and signal detection, which may require specific laboratory equipment and expertise. The need for such facilities and trained personnel restricts the widespread application of ELISA in resource-limited settings, remote areas, or during emergencies.

Cross-Reactivity and False Results

ELISA assays can be susceptible to cross-reactivity, leading to false-positive or false-negative results. Cross-reactivity occurs when antibodies or other components in the sample bind to non-specific targets, leading to incorrect interpretation of the results. This challenge is particularly pertinent with closely related coronaviruses or samples containing interfering substances. Efforts are underway to improve the specificity of ELISA assays through the selection of highly specific antigens, optimization of assay conditions, and thorough validation against well-characterized reference samples.

Future directions in ELISA for COVID-19 diagnostics

Multiplex ELISA Assays

A promising future direction is the use of multiplex ELISA assays. These assays enable the simultaneous detection of multiple antibodies or antigens in a single test, facilitating a comprehensive analysis of the immune response or identification of multiple pathogens. By incorporating multiple viral antigens or antibody isotypes into a single ELISA plate, researchers can gain a more thorough understanding of the immune response to COVID-19 and potentially distinguish it from other infections. Multiplex ELISA assays also offer advantages in terms of efficiency, cost-effectiveness, and conservation of time and sample volumes.

Point-of-Care Devices

A promising future direction for ELISA in COVID-19 diagnostics involves developing point-of-care devices. These devices aim to bring ELISA's diagnostic capabilities to bedside or field settings, eliminating the need for specialized laboratory facilities and reducing turnaround times for results. Portable point-of-care ELISA devices would enable rapid and convenient testing, facilitating timely decision-making and intervention. Efforts are underway to create miniaturized ELISA platforms that integrate all necessary components - such as sample processing, detection, and result interpretation - into a single device.

Integration With Other Diagnostic Platforms

Integrating ELISA with other diagnostic platforms shows promise for enhancing COVID-19 diagnostics. Combining ELISA with techniques like molecular diagnostics (e.g., PCR) or rapid antigen tests can provide complementary information and improve overall diagnostic accuracy. This integration enables a multi-modal approach to COVID-19 testing, leveraging the strengths of each platform to compensate for their limitations.

Discussion

ELISA has emerged as a cornerstone in COVID-19 diagnostics and research, offering a robust platform for detecting antibodies against SARS-CoV-2 with high specificity and sensitivity. The selection of viral antigens, optimization of assay formats, and validation processes are pivotal in ensuring accurate results.

Furthermore, ELISA has significantly contributed to the development and evaluation of therapeutic interventions for COVID-19. It has allowed researchers to assess the immune response of individuals to infection or vaccination, providing critical insights into the effectiveness of potential treatments, such as vaccines and monoclonal antibody therapies. The quantitative nature of ELISA has enabled researchers to measure antibody levels accurately, guiding the development and optimization of therapeutic strategies.

However, challenges exist in the field of ELISA for COVID-19 diagnostics. The requirement for specialized laboratory facilities and trained personnel can limit the widespread implementation of ELISA assays. Additionally, the potential for cross-reactivity and false results emphasizes the need for rigorous validation and quality control measures.

Despite its strengths, challenges such as the need for specialized facilities and the potential for cross-reactivity underscore ongoing efforts to innovate ELISA technology. Future directions, including multiplex assays and point-of-care devices, hold promise for expanding ELISA's utility, potentially overcoming current limitations and enhancing its role in global pandemic response strategies.

## Conclusions

In conclusion, ELISA has emerged as a crucial tool in the battle against the COVID-19 pandemic. Its ability to detect and quantify COVID-19 antibodies in patient samples has played a vital role in understanding the immune response to SARS-CoV-2 infection and evaluating the effectiveness of diagnostic and therapeutic interventions. ELISA has provided researchers with a reliable and accurate method for assessing the prevalence of COVID-19 antibodies in populations, aiding in epidemiological studies and surveillance efforts. By measuring antibody levels quantitatively, ELISA has helped monitor the effectiveness of vaccine campaigns and evaluate the duration of antibody protection, providing valuable data for public health strategies.

In summary, ELISA has demonstrated its significance in combating the COVID-19 pandemic by providing a reliable and quantitative method for detecting and analysing COVID-19 antibodies. Despite challenges such as cross-reactivity and the requirement for specialized facilities, ongoing innovations in multiplex assays and point-of-care devices promise to broaden ELISA's applicability and impact. As we continue to navigate the complexities of COVID-19 diagnostics, the evolution and refinement of ELISA methodologies underscore their pivotal role in understanding immune responses, monitoring vaccination efficacy, and informing public health strategies worldwide.
